# Vitamin D supplementation alleviates insulin resistance in prediabetic rats by modifying IRS-1 and PPARγ/NF-κB expressions

**DOI:** 10.3389/fendo.2023.1089298

**Published:** 2023-05-31

**Authors:** Desak Gede Budi Krisnamurti, Melva Louisa, Erni H. Poerwaningsih, Tri Juli Edi Tarigan, Vivian Soetikno, Heri Wibowo, Christian Marco Hadi Nugroho

**Affiliations:** ^1^ Department of Medical Pharmacy, Faculty of Medicine Universitas Indonesia, Jakarta, Indonesia; ^2^ Department of Pharmacology and Therapeutics, Faculty of Medicine, Universitas Indonesia, Jakarta, Indonesia; ^3^ Division of Endocrinology and Metabolism, Department of Internal Medicine, Dr. Cipto Mangunkusumo National Referral Hospital, Faculty of Medicine, Universitas Indonesia, Jakarta, Indonesia; ^4^ Department of Parasitology, Faculty of Medicine, Universitas Indonesia, Jakarta, Indonesia; ^5^ Research and Development Unit, Medika Satwa Laboratories, Bogor, Indonesia

**Keywords:** diabetes mellitus, high-fat diet, 25-hydroxyergocalciferol, inflammation, insulin resistance

## Abstract

**Background:**

Prediabetes is a condition of intermediate hyperglycemia that may progress to type 2 diabetes. Vitamin D deficiency has been frequently linked to insulin resistance and diabetes. The study aimed to investigate the role of D supplementation and its possible mechanism of action on insulin resistance in prediabetic rats.

**Method:**

The study was conducted on 24 male Wistar rats that were randomly divided into 6 rats as healthy controls and 18 prediabetic rats. Prediabetic rats were induced with a high-fat and high-glucose diet (HFD-G) combined with a low dose of streptozotocin. Rats with the prediabetic condition were then randomized into three groups of 12-week treatment: one group that received no treatment, one that received vitamin D3 at 100 IU/kg BW, and one group that received vitamin D3 at 1000 IU/kg BW. The high-fat and high-glucose diets were continuously given throughout the twelve weeks of treatment. At the end of the supplementation period, glucose control parameters, inflammatory markers, and the expressions of IRS1, PPARγ, NF-κB, and IRS1 were measured.

**Results:**

Vitamin D3 dose-dependently improves glucose control parameters, as shown by the reduction of fasting blood glucose (FBG), oral glucose tolerance test (OGTT), glycated albumin, insulin levels, and markers of insulin resistance (HOMA-IR). Upon histological analysis, vitamin D supplementation resulted in a reduction of the islet of Langerhans degeneration. Vitamin D also enhanced the ratio of IL-6/IL-10, reduced IRS1 phosphorylation at Ser307, increased expression of PPAR gamma, and reduced phosphorylation of NF-KB p65 at Ser536.

**Conclusion:**

Vitamin D supplementation reduces insulin resistance in prediabetic rats. The reduction might be due to the effects of vitamin D on IRS, PPARγ, and NF-κB expression.

## Introduction

Prediabetes is when an individual has above-average blood sugar levels but does not yet match the diagnostic criteria for diabetes. Prediabetes is not a disease in and of itself but rather an indicator of future health problems, including diabetes and cardiovascular disease (1). The World Health Organization instead called it “Intermediate Hyperglycemia.” At the same time, the American Diabetes Association referred to it as a “High-Risk State of Developing Diabetes” (2). Prediabetes is characterized by hyperinsulinemia which leads to insulin resistance. Eventually, chronic hyperinsulinemia will lead to beta cell dysfunction and favor the development of type 2 diabetes mellitus (2–4). Several strategies have been suggested for preventing diabetes in the prediabetic population. However, many few have been proven effective. No pharmacological intervention has been used explicitly to treat insulin resistance (4–6).

Recently, studies have linked vitamin D deficiency to diabetes pathogenesis (7–9). Several studies have shown that vitamin D deficiency may have a role in insulin resistance, yet the findings are still controversial. In some *in-vivo* and clinical studies, the lack of vitamin D levels has been associated with increased insulin resistance and impaired insulin production (7, 10–12). Vitamin D is suggested to promote insulin sensitivity and optimizes the activity of beta cells through several pathways. Vitamin D directly affects pancreatic beta cells by activating beta-cell calcium-dependent endopeptidases to release insulin (10, 12, 13).

In addition to vitamin D deficiency, a high-fat diet and sedentary lifestyle may produce adipocyte hypertrophy and hyperplasia, which aggravates hyperglycemia and hyperinsulinemia (14, 15). In a previous study in a mouse model with a high-fat diet, inflammatory insulin signaling markers were dysregulated. Chronic high fat intake will then be attributed to the development of insulin resistance (16).

Multiple studies have consistently shown reduced serum 25OHD concentrations in diabetic individuals. An analysis of the collective findings coming from multiple studies conducted to investigate the effectiveness of vitamin D supplementation in preventing type 2 diabetes revealed that compared to placebo, vitamin D supplementation reduced the risk of developing type 2 diabetes in people with prediabetes (17, 18). Despite the encouraging benefits of vitamin D, vitamin D supplementation in prediabetic and diabetic individuals has shown inconclusive outcomes in several studies (10, 19–21). Vitamin D supplementation’s mechanism of inhibiting insulin resistance in prediabetes has yet to be well understood. Thus, in the present study, we aimed to investigate the effect and mechanism of vitamin D supplementation in prediabetic rats on a high-fat, high-glucose diet.

## Methods

### Animals and treatments

The Health Research Ethics Committee of the Faculty of Medicine at Universitas Indonesia authorized this study (KET.701/UN2.F1/ETIK/PPM.00.02/2020). The experiments were carried out on male Wistar rats weighing 150–200 grams. The rats were acclimatized for 1 week in the Animal Research Facilities before the experiment. Six of the 24 rats were fed a standard diet (TestDiet™ 5012, Richmond, USA). 18 of the 24 rats were given a high-fat diet (TestDiet™ 58V8 rat chow, Richmond, USA) along with 20% glucose (HFD-G) in their drinking water to induce prediabetes. After three weeks, the rats in the high-fat, high-glucose groups were injected with 30 mg/kg BW streptozotocin. Seventy-two hours after streptozotocin injection, the rats were tested for oral glucose tolerance test (OGTT), fasting blood glucose (FBG), and 2-hour postprandial glucose (2H-PPG) concentrations. To confirm prediabetes conditions, all the rats had to meet 2 out of the 3 criteria: FBG of 100–125 mg/dL, OGTT of 140–199 mg/dL, and 2H-PPG prior to treatment randomization. The prediabetic rats were then randomly assigned to one of three groups of six: HFD-G + vehicle; HFD-G+ vitamin D3 100 IU/kg BW/day; or HFD-G+ vitamin D3 1000 IU/kg BW/day. The treatments were given for 12 weeks. The rat group given a standard diet continued to receive the same diet for an additional 12 weeks. At the end of the experiment, rats were sacrificed, blood samples were taken for biochemical testing, liver samples were used for western blot analysis, and pancreatic tissues were removed and fixed in 10% formal saline for histopathological analysis.

### Serum biochemical analysis

The current study measured blood glucose using serum rather than plasma. Even though serum produced lower values than plasma, the difference was not physiologically significant (22). The blood glucose concentrations were tested shortly after the blood was drawn. Blood glucose concentrations were quantified from serum samples on a Randox Glucose GOD-PAP GL 364 (Randox, UK) colorimetric kit. Blood glucose levels were calculated using the glucose oxidase technique described by Randox Laboratories Ltd (Ardmore, UK).

### Histological analysis

Pancreatic tissue samples were collected, dissected, and immediately fixed in 10% formalin for 24 hours, dehydrated using a graded alcohol series, cleaned in xylene, and finally embedded in paraffin. Tissue sections were stained with hematoxylin and eosin (H&E) for histopathological analysis (23). All areas were viewed using an OLYMPUS CX43 light microscope using a 400x magnification and shot with an OLYMPUS SC52 camera. The area of the Islet of Langerhans was counted using ImageJ, and two blind histopathologists examined all histological anomalies. The histological state of the pancreas was evaluated and then compared across the various treatment groups for damage and regeneration of pancreatic islet cells.

### Enzyme-linked immunoassay

The levels of insulin, glycated albumin, TNF-α, 25-hydroxycholecalciferol, IL-6, and IL-10 were quantified using enzyme-linked immunoassay kits according to the manufacturer’s instructions. Rat INS (Insulin) (Cat# ERINS), IL-6 (Cat# BMS625), and IL-10 (Cat# BMS629) ELISA kits were purchased from Thermo Scientific; rat glycated Albumin (Cat# No MBS1600353) and IRS1 (Cat No MBS9501484) ELISA kit from MyBioSource and 25-hydroxycholecalciferol (Cat No CSB-EL006431HV) ELISA kit from Cusabio.

### Western blot analysis

Proteins were isolated from liver tissue homogenates using 1x RIPA buffer. Moreover, the protein concentration was determined using a Coomassie Plus (Bradford) assay kit on a microplate reader spectrophotometer at 590 nm. The isolate was used for western blot analysis of protein expressions of NF-κB p65, PPARγ, and p-IRS1.

Primary antibodies used in the present study were obtained from Cell Signaling Technology (Beverly, MA): GAPDH (CST#2118), NF-κB p65 (CST#8242), phospho-NF-κB p65 (CST#3033), PPARγ (CST#2430), phospho-PPARγ (CST#2430), IRS1 (CST#2382), and p-IRS1 (Ser307) (CST#2381). Subsequently, 70 µg proteins were separated using 10% SDS-PAGE and transferred to a PVDF membrane. The quantity of protein used in the study corresponds with the study by Soetikno V. et al. (24). Blocking the membrane was done for 1.5 hours with 5% skimmed milk in phosphate buffer saline with Tween-20. After blocking, the membrane was incubated overnight at 4°C with a 1:1,000 dilution for all primary antibodies. Afterward, the membranes were washed in Tris-buffered saline with Tween-20 and incubated for 1 hour with secondary antibodies against Anti-rabbit IgG, HRP-linked Antibody (CST#7074) at a 1:5,000 dilution rate. Enhanced chemiluminescence (ECL) detection system reagents, Clarity Western (BioRad), were used to examine the targeted protein bands. ImageJ was used to evaluate the densitometry data (version 1.53a; National Institutes of Health). The bands presented were taken from the best acquisition and time in the ChemiDoc Imaging instrument (Biorad™).

### Statistical analysis

GraphPad Prism 9.4.1 software was used for the statistical analysis (GraphPad Software, Inc). The data were presented in the mean and standard error of the mean (SEM). Comparison between groups was analyzed using one-way ANOVA followed by Tukey’s *post hoc* test. A statistically significant difference was one with a p-value of less than 0.05.

## Results

### The effects of vitamin D supplementation in prediabetic rats

**Figure 1:**
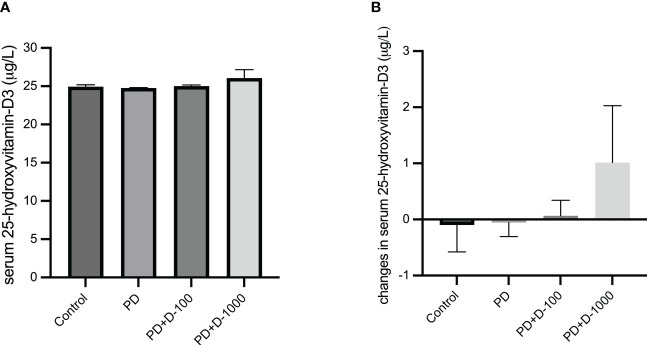
**(A)** Serum 25-hydroxy-vitamin D3 levels at the start of the treatment period; **(B)** changes in serum 25-hydroxy-vitamin D3 levels in healthy control or prediabetic rats after 12 weeks with no treatment or vitamin D 100 IU/kg BW/day or vitamin D 1000 IU/kg/BW/day.

The baseline serum 25-hydroxyvitamin-D3 (25-OH-D3) concentrations were measured before 12-week vitamin D3 supplementation. The results showed that the 25-OH-D3 average baseline levels in all four groups were below 30 μg/L, which indicates insufficient levels (**Figure 1A**). In prediabetic rats with no treatment, the 25-OH-D3 concentrations tend to decrease after twelve weeks. However, vitamin D3 supplementation may prevent the decrease of serum 25-OH-D3 levels in prediabetic rats given 100 IU/kg BW. Moreover, in prediabetic rats given 1000 IU/kg BW, there was a slight increase in serum 25-OH-D3 levels (**Figure 1B**).

**Figure 2:**
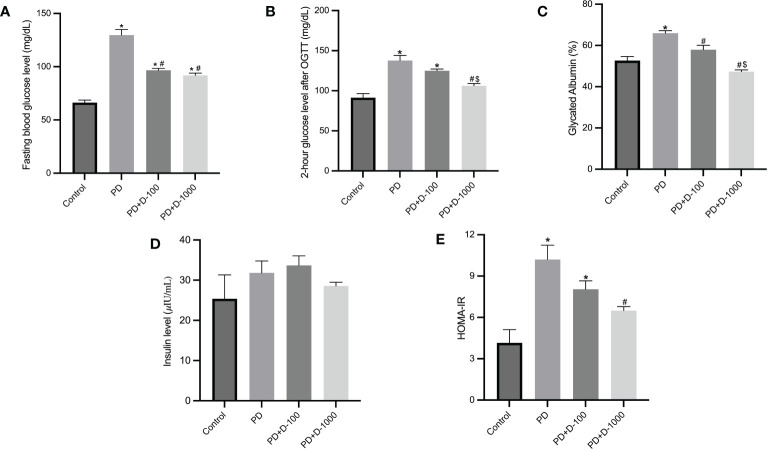
Markers of glucose control and insulin resistance in healthy control or prediabetic rats after 12 weeks with no treatment of vitamin D 100 IU/kg BW/day or vitamin D 1000 IU/kg/BW/day. **(A)** Fasting blood glucose; **(B)** 2-hour glucose level after oral glucose tolerance test (OGTT); **(C)** glycated albumin; **(D)** insulin level; **(E)** HOMA-IR. *: p<0.05 vs control; #: p<0.05 vs PD group; $: p<0.05 vs vitamin D-100 IU/kg BW/day group.

Hyperglycemia and hyperinsulinemia were shown in prediabetic rats compared to the control group. The status of insulin resistance was shown in HOMA-IR, and there was a substantial increase in HOMA-IR compared to the control group. Supplementation of vitamin D3 to prediabetic rats resulted in a considerable reduction in glucose control parameters and glycated albumin. As shown in HOMA-IR, insulin resistance was significantly decreased compared to the prediabetic group (**Figure 2**).

**Figure 3:**
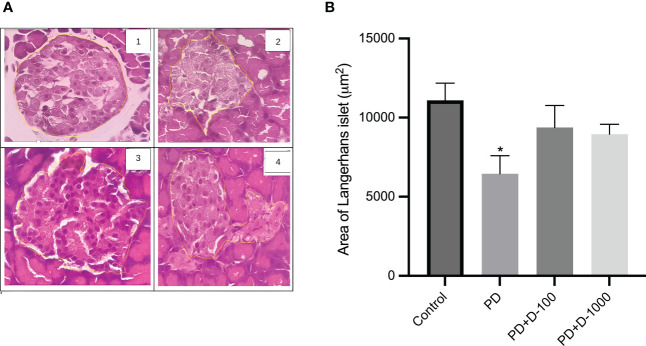
**(A)** Histology of the islet of Langerhans in the rat pancreas; **(B)** islet of Langerhans area in control or prediabetic rats after 12 weeks with no treatment or vitamin D 100 IU/kg BW/day or vitamin D 1000 IU/kg/BW/day. Magnification at 400x. The Islet of the Langerhans area was counted using the ImageJ analyzer. *: p<0.05 vs control.

Histopathology examinations of healthy control rat pancreas confirmed the islets of Langerhans’ regular shape. The prediabetic rat group induced with a high-fat diet showed pathological changes and cellular damage in the islets of Langerhans. Fat accumulation in pancreatic acinar cells is associated with pancreatic fibrosis and acinar cell damage. The pancreas of prediabetic rats also showed shrinkage of the islets of Langerhans, necrosis, and degeneration of the cells’ components (**Figure 3A**).

The supplementation of vitamin D3 at 100 IU/kg BW and 1000 IU/kg BW may minimize the damage in Langerhans’s islet and fatty pancreatic acinar cell atrophy. The pancreas of rats receiving vitamin D treatment has a virtually regular shape. The size of the islets of Langerhans is virtually restored to normal, while the number of fatty acinar cells is reduced (**Figure 3A**). As demonstrated in **Figure 3B**, there was a lower area of Langerhans islets in prediabetic rats compared to the control group. However, vitamin D3 supplementation tended to increase the area of Langerhans islets.

### Reduction of IRS-1 phosphorylation after vitamin D supplementation in prediabetic rats

**Figure 4:**
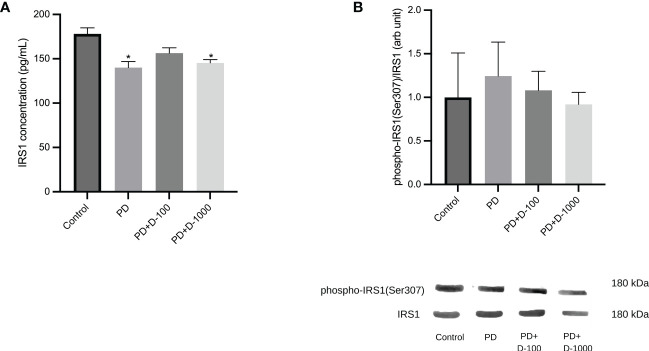
**(A)** Muscle IRS1 concentrations; **(B)** hepatic phospho-IRS1(Ser307)/IRS1 in healthy control or prediabetic rats after 12 weeks with no treatment or vitamin D 100 IU/kg BW/day or vitamin D 1000 IU/kg/BW/day. *: p<0.05 vs control.

There was a significant reduction in IRS1 concentrations in prediabetic groups compared to healthy control. However, vitamin D3 supplementation in prediabetic rats did not change the IRS1 concentrations (**Figure 4A**). Nevertheless, we observed a slight decrease in the phosphorylation of IRS1 after vitamin D3 supplementation at 100 IU/kg BW and 1000 IU/kg BW in the prediabetic group (**Figure 4B**).

### Modulation of serum inflammatory markers after vitamin D supplementation in prediabetic rats

**Figure 5:**
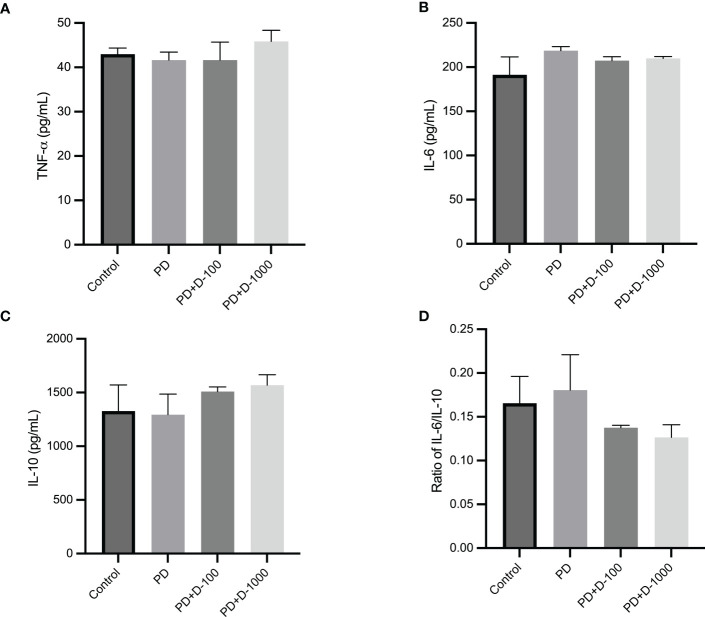
**(A)** Serum TNF-α concentration; **(B)** serum IL-6 concentration; **(C)** serum IL-10 concentration; **(D)** ratio of IL-6/IL-10 in healthy control or prediabetic rats after 12 weeks with no treatment or vitamin D 100 IU/kg BW/day or vitamin D 1000 IU/kg/BW/day.

The modulation of serum inflammatory markers in control, prediabetic, and prediabetic groups treated with vitamin D3 100 IU/kg BW or vitamin D3 1000 IU/kg BW was observed. There were no differences in TNF-α, IL-6, or IL-10 after 12 weeks of treatment. However, vitamin D3 supplementation tends to decrease the ratio of IL-6/IL-10 compared with the prediabetes group (**Figure 5**).

### Altered expressions of PPARγ and NF-κB phosphorylation after vitamin D supplementation in prediabetic rats

**Figure 6:**
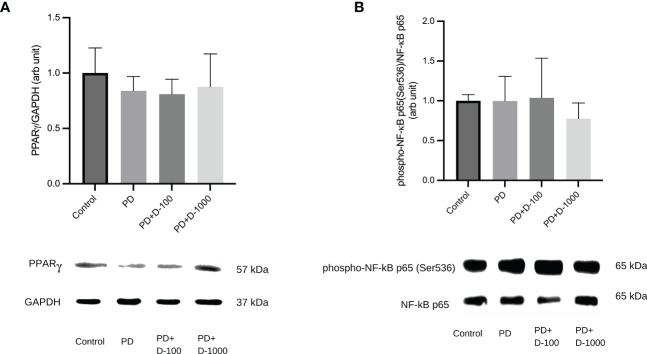
**(A)** Hepatic PPARγ/GAPDH expression; **(B)** Hepatic phospho-NF-κB p65(Ser536)/NF-κB in healthy control or prediabetic rats after 12 weeks with no treatment or vitamin D 100 IU/kg BW/day or vitamin D 1000 IU/kg/BW/day.

To illustrate the possible mechanism of vitamin D supplementation in prediabetic rats, we investigated PPARγ and NF-κB signaling by analyzing the expression of PPARγ and NF-κB p65 phosphorylation at Serine 536. We observed that supplementation of vitamin D3 at 100 IU/kg BW did little change in PPARγ expressions and NF-κB p65 phosphorylation. However, compared to the prediabetic group, vitamin D3 supplementation at 1000 IU/kg BW tends to increase PPARγ expression and NF-κB p65 phosphorylation (**Figure 6**).

## Discussion

In the present study, we examined the modulating effects of vitamin D supplementation on the molecular mechanism of insulin resistance in prediabetic rats. Our study showed that vitamin D3 administration improved glucose control and ameliorated insulin resistance in prediabetic rats. The modulation of the insulin signaling pathway and improved balance between proinflammatory, and anti-inflammatory cytokines contribute to reducing insulin resistance in prediabetic rats.

The prediabetic conditions in our study were induced by the chronic administration of a high-fat and high-glucose diet (HFD-G) in combination with a small dose of streptozotocin (30 mg/kg BW). The present model of prediabetes demonstrates impaired glucose homeostasis, as indicated by variations in glucose tolerance such as fasting blood glucose, OGTT, and insulin levels. No specific criteria for prediabetic conditions are currently available for rodents. However, the criteria used in the study paradigm are consistent with other studies (25, 26). HFD-G is widely used in the animal model of diabetes induction by inducing hyperglycemia and insulin resistance. Studies have shown that prolonged administration of a high-fat diet may inhibit the insulin receptor signaling pathway and trigger insulin resistance (27, 28). A high-fat diet will increase the formation of diacylglycerol (DAG) in the liver and muscles over time. Increased DAG in the liver activates protein kinase C (PKC), which phosphorylates serine residues on IRS1, blocking the phosphorylation of tyrosine residues. Insulin resistance develops because of a reduction in insulin-PI3K-Akt signaling. Reduced insulin-PI3K-Akt in muscle promotes reduced glucose absorption and a decrease in GLUT-4, both of which contribute to insulin resistance (28). In adipocytes, studies revealed a distinct connection between decreased insulin receptor expression and impairment of insulin signaling in adipocytes. One of the most critical roles is the activation of a miRNA (miR-128) in adipocytes, which causes mRNA instability of the insulin receptor (29, 30).

Studies showed impaired insulin signaling and secretion are linked to reduced 25-hydroxy-vitamin D3 concentrations in the blood in prediabetic individuals (7, 31). The current study showed that vitamin D3 supplementation might prevent the decrease of 25-hydroxy-vitamin D3 levels in the prediabetic group. Previous studies showed that a high-fat diet might increase vitamin D3 storage in the liver and adipose tissue, contributing to low serum 25(OH) D3 levels (32).

Based on findings linked to the role of vitamin D in insulin generation and glucose homeostasis, studies have demonstrated a causal relationship between vitamin D deficiency and diabetes mellitus (8, 9, 20, 21, 33, 34). Compared to the prediabetes group, vitamin D supplementation at 1000 IU/kg BW successfully lowered fasting blood glucose, plasma insulin, and insulin resistance, as shown by HOMA IR. In diabetic individuals, vitamin D levels were negatively associated with insulin resistance (HOMA-IR). Vitamin D deficiency is hypothesized to cause insulin resistance *via* several pathways, including increased proinflammatory cytokines, reduced insulin production by pancreatic beta cells, and decreased glucose absorption in peripheral tissues (20). Another study of diabetic rats given vitamin D supplementation (1000 IU and 2000 IU) for 45 days showed better glucose control and insulin resistance (35). Vitamin D indirectly impacts insulin secretion and interacts *via* β-cells to modulate extracellular calcium or calcium flow (21). Vitamin D may also activate calcium-dependent endopeptidase, which aids in the conversion of proinsulin to insulin (36).

Our findings were supported by histological examination using hematoxylin and eosin staining, which revealed a decrease in Langerhans islets as well as fatty pancreatic acinar cell atrophy while increasing the number of fatty acinar cells. For the histopathology analysis, we utilized the same strategy as earlier research that effectively reported pancreatic histology (37). The morphological differences between the negative and positive control groups, as well as the treatment group, were clearly visible using hematoxylin and eosin (H&E) staining. However, it will be beneficial for future research to add scan and image-based phenotypic analysis utilizing the cell painting approach.

Regarding to the vitamin D doses utilized in the study, were well below the hazardous quantity. Vitamin D toxicity is highly uncommon. The most common way to get vitamin D intoxication is by continuing to take very high dosages of vitamin D over an extended period. More than 150 µg/L may cause vitamin D intoxication and hypercalcemia in humans (38). In rats, the toxicity of vitamin D3 has been documented at extremely high dosages. Ali et al. reported vitamin D toxicity at a dose of 6,750 IU/rat/day, or equivalent to 27,000 IU/kg BW/day, while Chavhan et al. demonstrated toxicity at 2 mg/kg BW/day, or equal to 80,000 IU/kg BW/day (39, 40). In our study, the highest dose used was 1,000 IU/kg BW/daily, lower than those demonstrated in the studies of Ali et al. (39) and Chavhan et al. (40). Additionally, the highest plasma concentrations of vitamin D3 after treatment with vitamin D3 1,000 IU/kg BW were 31 µg/L.

In a rat model of type 2 diabetes mellitus with no vitamin D deficiency, vitamin D therapy was shown to reduce blood glucose levels by 40% (41). In addition to modulating calcium regulation in pancreatic beta cells, vitamin D3 directly impacts pancreatic beta cells *via* the binding to the vitamin D receptor (VDR) in the active form 1.25-hydroxy-vitamin D3. After binding to 1.25-hydroxyvitamin D3, VDR will interact with the vitamin D response element (VDRE), consequently leading to the insulin gene’s induced activation (11).

Our findings were consistent with those of Wahba et al., who discovered that vitamin D might improve oral glucose tolerance in prediabetic rats (42). The present study also showed that in both dosages studied, and vitamin D reduced glycated albumin levels. The link between vitamin D supplementation and glycated albumin levels in prediabetics has received little attention. Glycated albumin is a novel biomarker for monitoring short-term glycemic control due to its shorter half-life (2 to 3 weeks) compared to HbA1c (43).

Improvement of insulin resistance by vitamin D supplementation can be partially explained by reducing IRS1 phosphorylation at Serine 307. Insulin signaling is mediated by insulin receptor substrates 1 and 2 (IRS1 and IRS2), which regulate glucose homeostasis and energy metabolism. To date, the increased phosphorylation of IRS1 at serine 307 was considered the best available mechanism to understand the desensitization of insulin signaling (44). Phosphorylation of Ser307 in IRS1 limits insulin action by blocking connections with the insulin receptor (45). Our result was in line with a previous study in a diabetic rat model, which demonstrated that vitamin D supplementation for eight weeks and a high-fat diet reduced Ser307 phosphorylation of IRS1. Increased degradation of IRS1 causes impaired GLUT4 mobilization and decreased glucose uptake in the diabetic rat (46).

Multiple inflammatory responses are closely connected and play critical roles in developing insulin resistance and type 2 diabetes (47). Insulin resistance associated with obesity is characterized by chronic low-grade inflammation. There were increased proinflammatory cytokines and other bioactive compounds such as TNF-α, IL-1β, IL-6, or monocyte attractant protein-1 (MCP-1) (48). VDR, the receptor for 1.25-hydroxy-vitamin D3, is present in more than 38 different tissues and is known to regulate essential genes involved in bone metabolism, oxidative damage, chronic illnesses, and inflammation. Macrophages and dendritic cells express VDR constitutively, indicating that vitamin D likely plays a significant role in regulating the inflammatory response (49). Our study showed that vitamin D3 supplementation did not alter individual concentrations of proinflammatory cytokines (TNF-α, IL-6) and anti-inflammatory cytokines IL-10. Vitamin D3 may restore the balance between proinflammatory and anti-inflammatory cytokines, as shown by increasing the IL-10 levels and thus reducing the ratio of IL-6/IL-10.

The interaction between PPARγ and NF-κB is a signaling pathway that connects insulin resistance, metabolic syndrome, and inflammation (50, 51). PPARγ is a ligand-activated transcription factor that plays a crucial role in glucose homeostasis and adipocyte formation (52, 53). Several investigations have demonstrated that PPARγ may decrease inflammation by reducing NF-κB transcriptional activity by competing with p65 (51). The transcription factor NF-κB is an essential regulator of inflammation. It is necessary to produce proinflammatory cytokines such as IL-1β and IL-6 (54, 55). According to Ke et al., the inactivation of NF-κB p65 may modulate hepatic insulin sensitivity by elevating cAMP through PDE3B gene transcription suppression (55). Our data showed that 1000 IU/kg BW vitamin D3 supplementation enhanced PPARγ expression while decreasing NF-κB p65 phosphorylation at Ser536. NF-κB p65 phosphorylation at Ser536 is essential in inhibiting NF-κB transcription responses in toll-like receptor-activated macrophages, contributing to inflammation resolution (56).

## Conclusions

Our study indicated that vitamin D supplementation improves insulin resistance in prediabetic rats. Additionally, the decreased phosphorylation of IRS1 increased expression of PPARγ and reduced phosphorylation of NF-κB could be attributed to the attenuation of insulin resistance of vitamin D3. Therefore, vitamin D supplementation in a prediabetic state may prevent the progression of insulin resistance to diabetes.

## Data availability statement

The original contributions presented in the study are included in the article/supplementary materials. Further inquiries can be directed to the corresponding author.

## Ethics statement

The animal study was reviewed and approved by the ethics committee of The Faculty of Medicine Universitas Indonesia, Jakarta, Indonesia.

## Author contributions

DK, ML, TT, VS, and EP: study design; DK, ML, and VS: data analysis; DK, CN, and ML: funding; HW: acquisition. All authors contributed to the article and approved the submitted version.
